# Complications of Corneal Collagen Cross-Linking

**DOI:** 10.1155/2011/869015

**Published:** 2011-12-27

**Authors:** Shikha Dhawan, Kavita Rao, Sundaram Natrajan

**Affiliations:** Cornea and Anterior Segment, Aditya Jyot Eye Hospital, Mumbai 400031, India

## Abstract

Cross-linking of corneal collagen (CXL) is a promising approach for the treatment of keratoconus and secondary ectasia. Several long-term and short-term complications of CXL have been studied and documented. The possibility of a secondary infection after the procedure exists because the patient is subjected to epithelial debridement and the application of a soft contact lens. Formation of temporary corneal haze, permanent scars, endothelial damage, treatment failure, sterile infiltrates, and herpes reactivation are the other reported complications of this procedure. Cross-linking is a low-invasive procedure with low complication and failure rate but it may have direct or primary complications due to incorrect technique application or incorrect patient's inclusion and indirect or secondary complications related to therapeutic soft contact lens, patient's poor hygiene, and undiagnosed concomitant ocular surface diseases.

## 1. Introduction

 Keratoconus is a progressive, bilateral, often asymmetrical, and noninflammatory corneal ectasia. Prevalence of keratoconus is 1 : 2000 [1] and is usually diagnosed during the second and third decade of life. Currently available treatments for keratoconus (rigid contact lens, lamellar Keratoplasty, intacs) largely involve interventions which are done for tectonic, optical, or refractive purpose. Unfortunately, neither of those options treats the underlying cause of ectasia, and therefore cannot stop the progression of keratoconus.

 Corneal collagen cross-linking (CXL) based on the combined use of the photosensitizer riboflavin and UVA light of 370 nm was introduced by Wollensak et al. from Germany in 2003. CXL is the only available treatment directed at the underlying pathology in keratoconic cornea, which is stromal biomechanical and structural instability leading to progressive ectasia. CXL induces covalent inter- and intrafibrillar collagen cross-links creating an increase in biomechanical rigidity of human cornea by about 300%. The cross-linking effect is maximal only in the anterior stroma. Corneal collagen cross-linking (CXL) is currently under investigation to determine if it can slow, stabilize, or even possibly reverse the progression of corneal ectasia in patients with keratoconus [2]. The present paper is a review of literature on CXL complications.

## 2. Corneal Collagen Cross-Linking with Riboflavin and UVA

 The main indication for CXL in ophthalmology has been the management of corneal ectasia, such as halting the progression of keratoconus. In addition, CXL has been proposed as a treatment modality for iatrogenic keratectasia [3], infectious melting keratitis [4], and bullous keratopathy [5]. The latter application utilizes the antioedematous effect of cross-linkage on the stoma. CXL with riboflavin and UVA has been sequentially combined with other modalities, namely, intrastromal ring segments [6] and photorefractive keratectomy (PRK) [7] for the treatment of keratoconus.

 UVA irradiation can cause keratocyte and corneal endothelial cell destruction or death, as well as possible lens and retinal damage [8] as it has a toxic effect on cell viability, but there have been no reported complications on the endothelial cell count, lens, or retina due to the limitation of UVA transmission through the cornea [9]. It had also been suggested that CXL treatment be restricted to the anterior 250 *μ*m to 350 *μ*m of the stoma. Thus, CXL is not recommended for patients whose corneas are thinner than 400 *μ*m [10] because 85% to 90% of the UVA radiation is absorbed in the anterior 400 *μ*m of the cornea; the procedure should not harm the patient's corneal endothelium, lens, and retina [11].

## 3. Technique

 A standard CXL procedure begins with the administration of an anaesthetic, followed by debridement of the central 7 mm to 9 mm of the cornea to allow uniform diffusion of the riboflavin into the stroma [11]. Next, riboflavin 0.1% suspended in a dextran T500 20% solution is applied and allowed to permeate the cornea before UVA irradiation. UVA radiation of 370 nm wavelength and an irradiance of 3 mW/cm^2^ at a distance of 5.4 mm from the cornea is applied for a period of 30 min, delivering a dose of 5.4 J/cm^2^ [12]. Antibiotic eye drops are instilled as prophylaxis and a bandage contact lens is inserted, which is then removed at the followup visit once epithelial healing is complete.

## 4. Complications of CXL

 Several long-term and short-term complications of CXL have been studied and documented [13, 14] which may be direct or primary due to incorrect technique application or incorrect patient's inclusion or indirect or secondary complications related to therapeutic soft contact lens, patient's poor hygiene, and undiagnosed concomitant ocular surface diseases (dry eye, blepharitis, etc.).

### 4.1. Postoperative Infection/Ulcer

Debriding the corneal epithelium theoretically exposes the cornea to microbial infection. Bacterial keratitis has been reported 3 days following treatment in which scraping revealed an E. coli infection [15]. Acanthamoeba keratitis due to eye washing under tap water as the patient was unaware of a bandage contact lens being inserted has been reported [16]. Poor contact lens hygiene resulting in polymicrobial keratitis caused by *streptococcus salivarius, streptococcus oralis, *and coagulase-negative *staphylococcus *sp. has been reported recently [17]. A patient with no history of herpetic keratitis developed herpes simplex keratitis geographical ulcer and iritis five days after treatment [18].* Staphylococcus epidermidis *keratitis has also been reported 2 days after treatment [19]. Diffuse lamellar keratitis (stage 3) has been reported following treatment in a case of post-LASIK ectasia [20]. Severe keratitis with patient's contact lens and cornea scrapings positive for *pseudomonas aeruginosa *has also been reported recently [21]. Reactivated herpetic keratitis and neurodermatitis have also been reported following CXL [18, 22]. One study reported four cases of severe keratitis in a group of 117 keratoconic eyes treated with standard CXL [23].

Keratitis can occur following CXL because of presence of an epithelial defect, use of soft bandage contact lens, and topical corticosteroids in the immediate postoperative period. In cases of corneal infection after CXL, contact with the infectious agent likely occurred during the early postoperative period rather than during surgery because CXL not only damages keratocytes, but it also kills bacteria and fungi. This effect is used to advantage when CXL is performed for infectious keratitis.

### 4.2. Corneal Haze

In a recently published retrospective study of 163 eyes with grade I–III keratoconus, approximately 9% of the 127 patients developed clinically significant haze after 1-year followup. The subset of patients developing steroid resistant haze appeared to have more advanced keratoconus, as reflected in a lower mean corneal thickness and higher keratometry value of the apex compared with the control group [24]. An older age, grade III or IV keratoconus (according to krumeich's classification), and preoperative reticular pattern of stromal microstriae observed preoperatively by in vivo confocal microscopy [14] are considered risk factors for corneal haze post cross-linking. Advanced keratoconus should be considered at higher risk of haze development after CXL due to low corneal thickness and high corneal curvature [24].

After collagen cross-linking using riboflavin and UV-A, a lacunar honeycomb-like hydration pattern can be found in the anterior stroma with the maximum cross-linking effect, which is because of the prevention of interfibrillar cross-linking bonds in the positions of the apoptotic keratocytes [25]. The polygonal cross-linking network might contribute favorably to the biomechanical elasticity of the cross-linked cornea and to the demarcation of the anterior stroma after CXL on biomicroscopy [25], thus making lacunar edema a positive sign of efficient cross-linking. Another study documented stromal haze in 5 of 44 patients within 6 months of undergoing CXL. There has been a debate as to whether stromal haze is a normal finding after CXL because of its frequency [26].

Koller et al. [27] evaluated anterior stromal haze, which was graded on a scale used in cases after PRK [28]; the mean grade was 0.78, 0.18, and 0.06 at 1 month, 6 months, and 12 months, respectively. Previous confocal microscopy studies [26] report that a dense extracellular matrix compatible with clinical haze forms between 2 months and 3 months postoperatively.

The haze after CXL differs from the haze after PRK in stromal depth. Whereas haze after PRK is strictly subepithelial, haze after CXL extends into the anterior stroma to approximately 60% depth, which is on average equal to an absolute depth of 300 *μ*m. Haze after CXL is different in clinical character from haze after other procedures, such as excimer laser photorefractive keratomy. The former is a dustlike change in the corneal stroma or a midstromal demarcation line, whereas the latter has a more reticulated subepithelial appearance [29]. The haze may be associated with the depth of CXL into the stroma as well as the amount of keratocyte loss [26, 27].

Greenstein et al. [30] studied the natural course after CXL and found a significant postoperative increase in haze measured by both Scheimpflug densitometry and slit lamp assessment. The increase peaked at 1 month and plateaued between 1 month and 3 months. Between 3 months and 6 months, the cornea began to clear and there was a significant decrease in CXL-associated corneal haze which usually does not require treatment except for some low dose steroid medication in some cases. From 6 months to 1 year postoperatively, there continued to be a decrease in haze measurements. Typically late permanent scarring should be differentiated from the early postoperative temporary haze [31] which is often paracentral and compatible with good visual results. It may not be actually related to CXL itself but rather to the ongoing disease process and corneal remodeling.

Haze formation after CXL may be a result of back-scattered and reflected light, which decreases corneal transparency [32]. In vitro and ex vivo studies [33, 34] show that CXL leads to an immediate loss of keratocytes in the corneal stroma. In a confocal microscopy study, Mazzotta et al. [35] found that in eyes with keratoconus, activated keratocytes repopulated the corneal stroma starting at 2 months and that the repopulation was almost complete at 6 months. It is possible that these activated keratocytes contribute to the development of CXL-associated corneal haze. Other factors that may contribute to CXL-associated corneal haze include stromal swelling pressure changes [36], proteoglycan-collagen interactions [37], and glycosaminoglycan hydration [38]. Further study is needed to elucidate the pathophysiology of the development and time course of CXL-associated corneal haze.

### 4.3. Endothelial Damage

The endothelial damage threshold was shown to be at an irradiance of 0.35 mW/cm^2^, which is approximately twice compared with the 0.18 mW/cm^2^ that reaches the corneal endothelium when using the currently recommended protocol [10]. It may be due to a stromal thickness less than 400 *μ*m or incorrect focusing 3. If the procedure is done on a thinner cornea, it may lead to perforation 4. The recommended safety criteria must be observed because UV irradiation has potential to damage various intraocular structures.

### 4.4. Peripheral Sterile Infiltrates

Sterile corneal stromal infiltrates occur as a result of enhanced cell-mediated immunity to staphylococcal antigens deposited at high concentrations in areas of static tear pooling [39]. Sterile infiltration after CXL may be related to staphylococcal antigen deposition in areas of static tear pooling beneath the bandage contact lens [39]. 

### 4.5. Herpes Reactivation

Reactivation of HSV has been reported after emotional stress, trauma, fever, and laser surgery. These established clinical triggers are thought to be mediated by the adrenergic and sensory nervous systems. Exposure to UV light can also induce oral and genital herpes in humans and ocular herpes in animal models. Development of herpes keratitis and iritis after riboflavin-UVA treatment has been reported [18]. It seems that UVA light could be a potent stimulus to trigger/induce reactivation of latent HSV infections even in patients with no history of clinical herpes virus ocular infections. Significant corneal epithelial/stromal trauma or actual damage of the corneal nerves could be the mechanism of HSV reactivation. The use of topical corticosteroids and mechanical trauma caused by epithelial debridement may be additional risk factors. Prophylactic systemic antiviral treatment in patients with a history of herpetic disease after cross-linking with UVA might decrease the possibility of recurrence.

### 4.6. Treatment Failure

CXL failure is largely defined as keratoconic progression following treatment. One study of 117 eyes from 99 patients who underwent CXL documented a failure rate of 7.6% at one-year followup [27]. The results also indicated that 2.9% of eyes lost two or more lines of snellen visual acuity. Age older than 35 years, cornea thickness <400 *μ*m, and a preoperative CDVA better than 20/25 were identified as significant risk factors for complication. A high preoperative maximum keratometry reading was a significant risk factor for failure. Sterile infiltrates were seen in 7.6% of eyes and central stromal scars in 2.8%. The researchers concluded that changing the inclusion criteria may significantly reduce the complications and failures of CXL. Risk factors for CXL failure included a preoperative patient age of 35 years or older, spectacle-corrected visual acuity better than 20/25, and a maximum keratometry reading greater than 58.00 D [27].

## 5. Conclusion

Apart from haze and stromal hyperdensity after CXL with early or late onset as direct complication of the treatment, no other direct or primary complications of the procedure have been reported. Complications described in the literature are in the major part of indirect origin (infections, therapeutic contact lens, previous surgery (lasik), coexisting disorders of ocular surface, incorrect patient inclusion in the treatment, technical problems with UVA solid state emitter, wrong technique application, bad focusing, tilting, defocus, etc.). Therefore, only surgeons with sufficient experience in the management of corneal wound healing should perform this procedure. More studies are necessary to identify rare complications and to establish a list of indications regarding patient age, diagnosis, and the stage of keratectasia. The role of the UV light on the immune mechanisms of the cornea and its effect on corneal wound healing warrant further investigation. Repeat cross-linking treatments may become necessary in the long term. Considering that the turnover rate of stromal collagen fibres is several years, prospective studies with a followup of at least eight to ten years will be necessary.
